# Supplementary material for: Rational Design and Characterization of Trispecific Antibodies Targeting the HIV-1 Receptor and Envelope Glycoprotein
Source: Vaccines (Basel). 2023 Dec 23;12(1):19. doi: 10.3390/vaccines12010019 (PMC10819093; doi:10.3390/vaccines12010019)
Supplement: Supplementary file 1 [file vaccines-12-00019-s001.zip › vaccines-2730543-supplementary.pdf]

**Sequence of human heavy chain constant region**

ASTKGPSVFPLAPSSKSTSGGTAALGCLVKDYFPEPVTVSWNSGALTSGVHTFPAVLQSSG
LYSLSSVVTVPSSSLGTQTYICNVNHKPSNTKVDKRVEPKSCDKTHTCPPCPAPELLGGPS
VFLFPPKPKDTLMISRTPEVTCVVVDVSHEDPEVKFNWYVDGVEVHNAKTKPREEQYNS
TYRVVSVLTVLHQDWLNGKEYKCKVSNKALPAPIEKTISKAKGQPREPQVYTLPPSRDEL
TKNQVSLTCLVKGFYPSDIAVEWESNGQPENNYKTTPPVLDSDGSFFLYSKLTVDKSRWQ
QGNVFSCSVMHEALHNHYTQKSLSLSPGK*

**Sequence of human light chain constant region**

RTVAAPSVFIFPPSDEQLKSGTASVVCLLNNFYPREAKVQWKVDNALQSGNSQESVTEQD
SKDSTYSLSSTLTLSKADYEKHKVYACEVTHQGLSSPVTKSFNRGECS*

**iMab-heavy chain**

QVQLQQSGPEVVKPGASVKMSCKASGYTFTSYVIHWVRQKPGQGLDWIGYINPYNDGT
DYDEKFKGKATLTSDTSTSTAYMELSSLRSEDTAVYYCAREKDNYATGAWFAYWGQGTLV
TVSSASTKGPSVFPLAPCSRSTSESTAALGCLVKDYFPEPVTVSWNSGALTSGVHTFPAVLQ
SSGLYSLSSVVTVPSSSLGTKTYTCNVDHKPSNTKVDKRVESKYG

**iMab-Light chain**

DIVMTQSPDSLAVSLGERVTMNCKSSQSLLYSTNQKNYLAWYQQKPGQSPKLLIYWASTR
ESGVPDRFSGSGSGTDFTLTISSVQAEDVAVYYCQQYYSYRTFGGGTKLEIKRTVAAPSVFI
FPPSDEQLKSGTASVVCLLNNFYPREAKVQWKVDNALQSGNSQESVTEQDSKDSTYSLSS
TLTLSKADYEKHKVYACEVTHQGLSSPVTKSFNRGEC

**10E8-heavy chain**

EVQLVESGGGLVKPGGSLRLSCAASGFTFSNAWMSWVRQAPGKGLEWVGRIKSKTDGGT
TDYAAPVKGRFTISRDDSKNTLYLQMNSLKTEDTAVYYCARTGKYYDFWSGYPPGEEYF
QDWGRGTLVTVSSASTKGPSVFPLAPSSKSTSGGTAALGCLVKDYFPEPVTVSWNSGALT
SGVHTFPAVLQSSGLYSLSSVVTVPSSSLGTQTYICNVNHKPSNTKVDKRVEPKSCDK

**10E8-Light chain**

SYELTQETGVSVALGRTVTITCRGDSLRSHYASWYQKKPGQAPILLFYGKNNRPSGVPDRF
SGSASGNRASLTISGAQAEDDAEYYCSSRDKSGSRLSVFGGGTKLTVLSQPKAAPSVTLFP
PSSEELQANKATLVCLISDFYPGAVTVAWKADSSPVKAGVETTTPSKQSNNKYAASSYLSL
TPEQWKSHRSYSCQVTHEGSTVEKTVAPTECS

**PGT121-heavy chain**

QVQLQESGPGLVKPSETLSLTCTVSGGSISSYYWSWIRQPPGKGLEWIGYIYYSGSTNYNP
SLKSRVTISVDTSKNQFSLKLSSVTAADTAVYYCARTQQGKRIYGVVSFGDYYYYYYMDV
WGKGTTVTVSSASTKGPSVFPLAPSSKSTSGGTAALGCLVKDYFPEPVTVSWNSGALTSG
VHTFPAVLQSSGLYSLSSVVTVPSSSLGTQTYICNVNHKPSNTKVDKRVEPKSCDKT

**PGT121-Light chain**

SYVLTQPPSVSVAPGQTARITCGGNNIGSKSVHWYQQKPGQAPVLVVYDDSDRPSGIPERF

SGSNSGNTATLTISRVEAGDEADYYCQVWDSRGPTNWVFGGGTKLTVLGQPKAAPSVTLF
PPSSEELQANKATLVCLISDFYPGAVTVAWKADSSPVKAGVETTTPSKQSNNKYAASSYLS
LTPEQWKSHKSYSCQVTHEGSTVEKTVAPTECS

**PGDM1400-heavy chain**

QAQLVQSGPEVRKPGTSVKVSCKAPGNTLKTYDLHWVRSVPGQGLQWMGWISHEGDK
KVIVERFKAKVTIDWDRSTNTAYLQLSGLTSGDTAVYYCAKGSKHRLRDYALXDDDGAL
NWAVDVDYLSNLEFWGQGTAVTVSSASTKGPSVFPLAPSSKSTSGGTAALGCLVKDYFPE
PVTVSWNSGALTSGVHTFPAVLQSSGLYSLSSVVTVPSSSLGTQTYICNVNHKPSNTKVDK
KVEPKSCD

**PGDM1400-light chain**

DFVLTQSPHSLSVTPGESASISCKSSHSLIHGDRNNYLAWYVQKPGRSPQLLIYLASSRASG
VPDRFSGSGSDKDFTLKISRVETEDVGTYYCMQGRESPWTFGQGTKVDIKRTVAAPSVFIF
PPSDEQLKSGTASVVCLLNNFYPREAKVQWKVDNALQSGNSQESVTEQDSKDSTYSLSST
LTLSKADYEKHKVYACEVTHQGLSSPVTKSFNRGEC

**Recombinant CD4 protein** (aa 26-396, sequence as following:
KKVVLGKKGDTVELTCTASQKKSIQFHWKNSNQIKILGNQGSFLTKGPSKLNDRADSRRS
LWDQGNFPLIIKNLKIEDSDTYICEVEDQKEEVQLLVFGLTANSDTHLLQGQSLTLTLESPP
GSSPSVQCRSPRGKNIQGGKTLSVSQLELQDSGTWTCTVLQNQKKVEFKIDIVVLAFQKA
SSIVYKKEGEQVEFSFPLAFTVEKLTGSGELWWQAERASSSKSWITFDLKNKEVSVKRVT
QDPKLQMGKKLPLHLTLPQALPQYAGSGNLTLALEAKTGKLHQEVNLVVMRATQLQKNL
TCEVWGPTSPKLMLSLKLENKEAKVSKREKAVWVLNPEAGMWQCLLSDSGQVLLESNIK
VLPTWSTPVQP)

**CCR5 protein** (aa 1-352, sequence as following:
MDYQVSSPIYDINYYTSEPCQKINVKQIAARLLPPLYSLVFIFGFVGNMLVILILINCKRLKS
MTDIYLLNLAISDLFFLLTVPFWAHYAAAQWDFGNTMCQLLTGLYFIGFFSGIFFIILLTIDR
YLAVVHAVFALKARTVTFGVVTSVITWVVAVFASLPGIIFTRSQKEGLHYTCSSHFPYSQY
QFWKNFQTLKIVILGLVLPLLVMVICYSGILKTLLRCRNEKKRHRAVRLIFTIMIVYFLFWA
PYNIVLLLNTFQEFFGLNNCSSSNRLDQAMQVTETLGMTHCCINPIIYAFVGEKFRNYLLV
FFQKHIAKRFCKCCSIFQQEAPERASSVYTRSTGEQEISVGL)

**Whole genome sequence of HIV Shenzhen BC**

strainTAGTGGCGCCCGAACAGGGACCTTGAAAGCGAAAGTAGAACCAGAGGAGATCTC
TCGACGCAGGACTCGGCTTGCTGAAGTGCACTCGGCAAGAGGCGAGAGCGGCGGCTG
GTGAGTACGCCAATTTTATTTGACTAGCGGAGGCTAGAAGGAGAGAGATGGGTGCGAG
AGCGTCAGTATTAAGAGGCGGAAAATTAGATAAATGGGAAAAAATTAGGTTAAGGCCA
GGGGGAAAGAAAAAATATAGGCTAAAACACCTAGTATGGGCAAGCAGGGAGCTGGAA
AAATTTGCACTTAACCCTGACCTTTTAGAGACAGCAGAAGGCTGTAAACAAATAATAA
AACAGTTACACCCAGCTCTTAAGACAGGAACAGAAGAACTTAAATCATTATTCAACTT
AGTAGCAGTTCTCTATTGTGTACATTCAAATATAGATGTGAAAGACACCAAAGAAGCCT
TAGACAAGATAGAGGAAGAACAAAACAAAATTCAGCAAAAAACACAGCAGGCAAAG

GAGGCTGACAAAAAGGTCAGTCAAAATTTTCCTATAGTGCAGAATATCCAAGGGCAAA
TGGTACATCAGCCCATATCACCTAGAACYTTAAATGCATGGGTAAAAGTGGTAGAAGA
AAAGGCTTTTAGCCCAGAAGTAATACCCATGTTTTCAGCATTATCAGAAGGAGCCACCC
CACAAGATTTAAACACCATGCTAAACACAGTGGGGGGACATCAAGCAGCCATGCAAAT
ATTAAAAGACACCATCAATGAAGAAGCTGCAGAATGGGATAGATTACATCCAGTACATG
CAGGGCCTGCTGCACCAGGCCAAATGAGAGAACCAAGGGGAAGTGACATAGCAGGA
ACTACCAGTACCCTTCAGGAACAAATAGCATGGATGACGAGTACTCCACCTGTTCCAG
TAGGAGACATCTATAAAAGGTGGATAATTCTGGGATTAAATAAAATAGTAAGAATGTATA
GCCCCACCAGCATTCTGGACATAAAACAAGGGCCAAAGGAACCCTTTAGAGAYTATGT
AGACAGGTTCTTTAAAACTTTGAGAGCAGAACAAGCTACACAAGATGTAAAAAATTGG
ATGACAGACACCTTGTTGGTCCARAATGCGAACCCAGATTGTAAGACCATTTTAAGAG
CATTAGGACCAGGGGCTTCAATAGAAGAAATGATGACAGCATGTCAGGGAGTGGGAG
GACCTAGCCATAAAGCAAGAGTGTTGGCTGAGGCAATGAGCCAAACAAACAATGCCA
TAATGATGCAGAGAAGCAATTTTAAAGGTCCTAAAAGAATTATTAAATGTTTCAACTGT
GGCAAGGAAGGGCACATAGCCAAAAATTGTAGGGCCCCTAGAAAAAAGGGCTGTTGG
AAATGTGGGAAAGAAGGACACCAAATGAAAGACTGTACTAATGAGAGACAGGCTAAT
TTTTTAGGGAAAATCTGGCCCTCCCACAAGGGAAGGCCAGGGAATTTTCTTCAGAGCA
GGCCAGAACCAACAGTCCCAACAGCCCCACCAGAGGAGAGCTTCAGGTTTGGGGAA
GAGATGACAACTCCATCTCAAAAGCAGGAGCCAGTAACTTCCCTCAAATCACTCTTTG
GCAACGACCCCTTGTTACCATAAGGATAGGGGGGCAATTAAAAGAAGCTCTATTAGATA
CAGGAGCAGATGATACAGTATTAGAAGAAATGAATTTGCCAGGGAAATGGAAACCAAA
AATGATAGGGGGAATTGGAGGTTTTATCAAAGTAAGACAGTATGAAGAGRTACCCATAG
AAATCTGTGGACACAAAGTTATAGGTACAGTATTAATAGGACCTACACCTGTTAACATA
ATTGGAAGAAATCTGTTAACTCAGCTTGGTTGTACTTTAAATTTTCCAATCAGTCCTATT
GAAACTGTACCAGTAAGACTAAAGCCAGGAATGGATGGCCCAAAGGTTAAACAATGG
CCATTAACAAAAGAGAAAATAGAAGCATTAACAGAAATTTGTAAAGAAATGGAAAAG
GAAGGAAAAATTACAAAAATTGGGCCTGAAAATCCATACAACACTCCAATATTTGCTAT
AAAAAAGAAAGACAGTACTAAGTGGAGAAAATTAGTAGATTTCAGGGAACTCAATAA
AAGAACTCAAGATTTTTGGGAGGTTCAATTAGGAATACCACACCCAGCAGGATTAAAA
AAGAAAAAATCAGTGACAGTGCTGGATGTGGGGGATGCATATTTTTCAGTTCCTTTAGA
TGAAGACTTCAGAAAATATACTGCATTCACCATACCTAGTGTAAACAATGRAACACCAG
GGATTAGGTATCAGTACAATGTACTTCCACAGGGATGGAAGGGATCACCAGCAATATTT
CAAAGYAGCATGACAAAAATCTTAGAGCCTTTTAGAAAACAAAATCCAGACATAGTCA
TCTATCAATACATGGATGATTTGTATGTAGGATCTGACTTAGAAATAGGACAGCATAGAA
CAAAAATAGAGGAACTGAGAGAACACTTGTTGAGGTGGGGATTTACCACACCAGACA
AGAAACATCAGAAAGAACCTCCATTTCTTTGGATGGGGTATGAACTCCATCCCGACAA
ATGGACAGTACAGCCTATACATCTGCCAGAACAAGATAGCTGGACTGTCAATGATATAC
AAAAGTTAGTGGGAAAATTAAACTGGGCAAGTCAGATTTATCCTGGAATTAAAGTAAG
GCAACTTTGCAAACTCCTTAGGGGGGCCAAAGCACTAACAGACATAGTACCACTAACT
GAAGAAGCAGAATTAGAATTGGCAGAAAACAGGGAAATYCTAAAAGAACCAATACAT
GGAGCATACTATGACCCATCAAAAGACTTGATAGCTGAGATACAGAAACAGGGGCAGG
ACCAATGGTCATATCAGATTTATCAAGAACCATTCAAAAATCTAAAAACAGGAAAGTAT
GCAAAAATGAGGACTGCCCACACTAATGATGTAAGACAATTAACAGAAGCTGTGCAGA
AGATAGCCATGGAAAGCATAGTAATATGGGGAAAGACTCCTAAATTTAGACTACCCATC

CAAAAGGAGACATGGGAGACATGGTGGACAGACTATTGGCAAGCCACCTGGATTCCT
GAGTGGGAATTTGTTAATACCCCTCCCTTAGTAAAATTATGGTACCAGCTGGAAAAAGA
CCCCATAGCAGGAGCAGAAACTTTCTATGTAGATGGAGCAGCTAATAGGGAAACTAAA
ATAGGAAAAGCAGGGTATGTTACTGACAGAGGAAGGAAAAAAGTTGCTACTCTAAATG
AAACAACAAATCAGAAAACTGAATTGCATGCAATCTGTCTAGCGTTGCAAGATTCAGG
ATCAGAAGTAAACATAGTAACAGATTCACAGTATGCATTAGGGATCATTCAAGCACAAC
CAGATAAGAGTGACTCAGAGTTAGTTAACCAAATAATAGAACAATTAATAAAAAAGGA
AAGAGTCTACCTGTCATGGGTACCAGCACATAAAGGAATTGGAGGAAATGAACAAGTA
GATAAATTAGTAAGTAGTGGTATCAGGAAAGTATTATTTCTAGATGGAATAGATAAAGCT
CAAGAAGAGCATGAAAGGTATCACAGCAATTGGAGAGCAATGGCTAGTGATTTTAATC
TGCCACCCGTAATAGCAAAAGAAATAGTGGCTAGCTGTGATAAATGTCAGTTAAAAGG
AGAAGCCATGCATGGACAAGTAGACTGTAGTCCAGGGATATGGCAATTAGATTGTACCC
ATYTAGAAGGAAAGATTATCCTGGTAGCAGTCCATGTAGCCAGTGGCTACATAGAAGC
AGAGGTTATTCCAGCAGAAACAGGACAAGAGACAGCATACTTTATACTAAAATTAGCA
GGAAGATGGCCAGTCAAAGTAATACATACAGACAATGGTAGTAATTTCACCAGTGCTG
CAGTTAAGGCAGCCTGTTGGTGGGCAGGCCTCCAACAGGAATTTGGAATTCCCTACAA
TCCCCAAAGTCAGGGAGTAGTAGAATCCATGAATAAAGAATTAAAGAAAATTATAGGG
CAGGTAAGAGATCAAGCTGAACACCTTAAGACAGCAGTACAAATGGCAGTATTCATTC
ACAATTTTAAAAGAAAAGGGGGGATTGGGGGGTACAGTGCAGGGGAGAGAATAATAG
ACATAATAGCAACAGACATACAAACTAAAGAATTACAAAAACAAATTACAAAAATTCA
AAATTTTCGGGTTTATTWTCAGRGACAGCAGAGACCCCATTTGGAAAGGACCAGCCAA
ACTACTCTGGAAAGGTGAAGGGGCAGTAGTAATACAAGATAATAGTGACATAAAGGTA
GTACCAAGGAGGAAAGCAAAAATCATTAAGGGACTATGGMAAACAGATGGCAGGYG
MTGATTGTGTGGCAGGTAGACAGGATGAAGATTAGAACATGGAATAGTTTAGTAAAAC
ACCATATGTATGTTTCCAGAAGAGCTAATGGATGGTTCTACAGACATCATTATGAGAGC
AGACATCCGAAAATAAGTTCAGAAGTACACATCCCAATGGGAGAAGCTAAATTAGTAA
TAAAAACATATTGGGGGTTGCAAACAGGAGAAAGAGATTGGCATTTGGGACATGGAGT
TTCCATAGAATGGAGATTCAAAAGATATAGCACACAAATAGAACCTGGTCTAGCAGACC
AACTAATTCATTTATATTATTTTGATTGTTTTGCAGACTCTGCTATAAGGAGAGCCATATT
AGGACATATAGTAATTCCTAGGTGTGACTATCAAGCAGGGCATAATAAGGTAGGATCTC
TACAATATTTGGCACTGACAGCATTGATAAAACCAAAAAGGATAAAGCCACCTCTGCCT
AGTGTTAAGAAACTAGTAGAAGATAGATGGAACAAGCCCCAGAAGACCAGGGGCCAC
AGAGGGAACCATTCAATGGATGGACAATAGAGCTTCTAGAGGAGCTCAAGCAGGAAG
CTGTCAGACACTTTCCTAGACCATGGCTTCATGGCTTAGGACAATACATCTATGAAACA
TATGGGGATACTTGGGCAGGGGTGGAAGCTATAATAAGGATTCTACAACAACTGCTGTT
TGTCCATTTCAGAATTGGGTGTCAGCATAGCAGAATAGGCATTTTGAGACAGAGAAGA
ACAAGAAATGGAGCCAGTAGATCCTAAATTAGAGCCTTGGAAGCATCCAGGAAGTCAG
CCTAAGACTGCTTGTACAAATTGCTATTGTAAAAGATGCTGCTTACACTGCCAAGTTTG
TTTCATGCAAAAAGGCTTAGGAATCTTCTATGGCAGGAAGAAGCGAAGGCAACGACG
AAGACCTCCTCAGAGCAGTGAGGATCATCAAAATTCTATACCAAAGCAGTAAGTAGTA
AATGTAATGCAAGCTTTAGCCATTTTTGCAATAGTAGCCTTAATAGTAGTAGGAATAATA
GCAATAGTTATATGGACAATAGTACTCATAGAATATAGAAAAATATTAAGACAGAGAAA
AATAGATAGGTTAATTGATAGAATAAGAGAAAGAGCAGAAGACAGTGGCAATGAGAGT
GACGGGGATCAAGAAGAATTGGCATTTTTGGAAATGGGGCACCTTGCTCCTTGGAATG

TTGATGATCTGTAGTGCTGCAGCAAACTTGTGGGTCACAGTCTATTATGGGGTACCTGT
ATGGAAAGAAGCAACCACAACTTTATTTTGTGCATCAGATGCTAAATCATATGATACAG
AGGTACATAATGTCTGGGCTACACATGCCTGTGTACCCACAGACCCTAACCCACAGGA
AATAGTCTTGGCAAATGTAACAGAAAATTTTAACATGTGGAAAAATGATATGGTAAATC
AGATGCATGAAGATGTAATCAGTTTATGGGATCAAGGCCTAAAGCCATGTGTAAAGTTG
ACCCCACTCTGTGTCACTTTAAATTGTACAGATGTTAGGAATGGTACAAAGAATACCAG
TACAAAGAATACCAGTACTATTAGCAATACTAACAGTACTATGAAAAATTGCTCTTTCA
ATATAACCACAGTATTAAGAGATAAGAAGCAGCAAGTGTATGCACTTTTTTATAAACTT
GATATAGTACCACTTAATGATGATAATTCTAGTGATCCTAATGGTGAGTATAGATTAATAA
ATTGTAATACCTCAACCATGACGCAAGCCTGTCCAAAGATCTCATTTGATCCAATTCCTA
TACACTATTGCACTCCTGCTGGTTATGCACTTCTCAAGTGTAATGATGAGAATTTCAATG
GAACAGGACCATGCCCTAATGTTAGTTCAGTACAATGTACACATGGGATTAAGCCAGTG
GTATCAACTCAACTACTATTAAATGGTAGTCTAGCAGAAGATGAAATAGTAGTTAGATCT
GAAAATCTGACAAACAATGTCAAAACAATAATAGTAAACCTTAATAAATCTGTAGAAAT
TGTATGTACAAGACCTAACAATAATACAAGAAAAAGTATAAGGATAGGACCAGGACAA
ACATTTTATGCAACAGGAGACATAATAGGAGACATAAGACAAGCACATTGTAACATTAG
TGGTTGGCAAGACATGTTACATAATGTAAGTAAAAAATTAGCAAAACTCTACCCAAATA
AAACAATAATATTTGAACCAGCCTCAGGAGGGGACTTAGAAATTACCACACATAGCTTT
AATTGTAGAGGGGAATTTTTCTATTGCAATACATCAGGCCTGTTTAATAGTACATTCAAT
GGTACATTTACTTTTAATAATACATCCAATGATACAGGAAAGAATGGGAGCATCACAATC
CCATGCAGAATAAAGCAAATTGTAAATATGTGGCAGGAGGTAGGACGAGCAATGTATG
CTCCTCCCATTGCAGGAAACATAACATGCAAGTCAAATATCACAGGACTGTTATTAGTA
CGTGATGGAGGGACTGGTAGAGACTCAAATAATACAGAGAAATTCAGGCCTGGAGGA
GGAGATATGAGGGACAATTGGAGAAGTGAATTATATAAATATAAAGTGGTGGAAATTAA
GCCATTGGGAATAGCACCCACCCAAGCAAAAAGGAGAGTGGTGGGAAAAGAAAAAA
GAGCAGTGGGAATAGGAGCTGTGTTCCTTGGGTTCTTGGGAGTAGCAGGAAGCACTAT
GGGCGCGGCGGCAATGACGCTGACGGTACAGGCCAGACAATTGCTGTCTGGTATAGTG
CAACAGCAAAACAATTTGCTGAGAGCTATAGAGGCGCAACAGCATATGTTGCAACTCA
CAGTCTGGGGCATTAAACAGCTCCAAACAAGAGTCCTGGCTATAGAAAGATACCTAAA
GGATCAACAGCTCCTAGGGATTTGGGGCTGCTCTGGAAAACTCATCTGCACCACTGCA
GTACCTTGGAACACCAGTTGGAGTAACAAATCTGAACAGGACATTTGGAATAACCTGA
CCTGGATGCAATGGGATAAAGAAATTAATAATTACACAAACACAATATACAGTTTGCTT
GAAGAGGCGCAGAACCAGCAGGAAAGAAATGAGAAAGATCTATTAGCATTGGACAGG
TGGGAAAGTCTATGGAATTGGTTTAACATAACGAATTGGCTGTGGTATATAAAAATATTC
ATAATGATAGTAGGAGGCTTGATAGGCTTAAGAATAATTTTTGCTGTGCTCTCTATAGTG
AATAGAGTTAGGCAGGGATACTCACCCTTGTCATTGCAGACCCTTATCCCGAACCCAGA
GGGACCCGACAGGCTCAGAAGAATCGAAGAAGAAGGTGGAGAGCAAGACAGAGACA
GATCCATTCGATTAGTGACCGGATTCTTAGCACTTGCCTGGGACGACCTGCGGAACCTG
TGCCTCTTCAGCTACCACCGATTGAGAGACTTTATATTGGTGACAGCGAGAGTGGTGG
AACTTCTGGGACGGAACAGCCTCAGGGGACTACAGAGGGGGTGGGAAATCCTTAAAT
ATCTAGGAAGCCTTGTGCAGTACTGGGGGCAGGAGCTAAAAAAGAGTGCTGTTAGTCT
GCTTGATACCATAGCAATAGTAGTAGCTGAAGGAACAGATAGGATTATAGAAGTAGGAC
AAAGAATTTGTAGAGCTATCTACAACATACCTAGAAGAATAAGACAGGGCTTTGAAGC
AGCTTTGCAATAAAATGGGGGCCAAGTGGTCAAAAGGATGCCCTGCTGTAAGGGAAA

GAATGAGACGAACTCAGCCAGCAGCAGATAGGAGGGAAAGAAGGAGACAAACTGAA
CCAGCAGCAGATGGGGTGGGAGCAGCATCTCGAGACCTGGAAAGACATGGAGCAATA
ACAAGTAGCAATACAGCAGCTACTAATGCTGATGTTGCATGGTTGGAAGCCCAACAGG
AGGAGGAGGAGGAAGTGGGTTTTCCAGTCAGACCTCAGGTACCTTTAAGGCCAATGA
CTTCAAGGGTAGCTTTTGATCTTAGCTTCTTTTTAAAAGAAAAGGGGGGACTGGATGG
GTTAGTTTACTCTAAGAAAAGGCAAGAGATCCTTGATTTGTGGGTCTATCACACACAAG
GCTTCTTCCCTGATTGGGACAACTACACACCAGGGCCAGGGACCAGGTTCCCACTGAC
TTTTGGGTGGTGCTTCAAGCTAGTACCAGTTGACCCAAAGGAAGTAGAAGAGATCACC
GCAGGAGAAGACAACTGCTTGCTACACCCTGTGTGCCAGCATGGAATGGAGGATGAG
CACGGAGAAGTATTAATATGGAAGTTTGACAGCATGCTAGCACGCAGACACATGGCCC
GCGAGCTACATCCGGAGTTTTACAAAGACTGCTGACACAGAAGGGACTTTCCGCGGG
GACTTTCCACTGGGGCGTGCTGGGAGGTGTGGTCTGGGCGGGACTGGGAGTGGTCAA
CCCTCAGATGCTGCATATAAGCAGCTGCTTTTCGCCTGTACTGGGTCTCTCTAGTCAGA
CCAGATTTGAGCCTGGGAGCTCTCTGGCTGCTAGGGAACCCACTGT

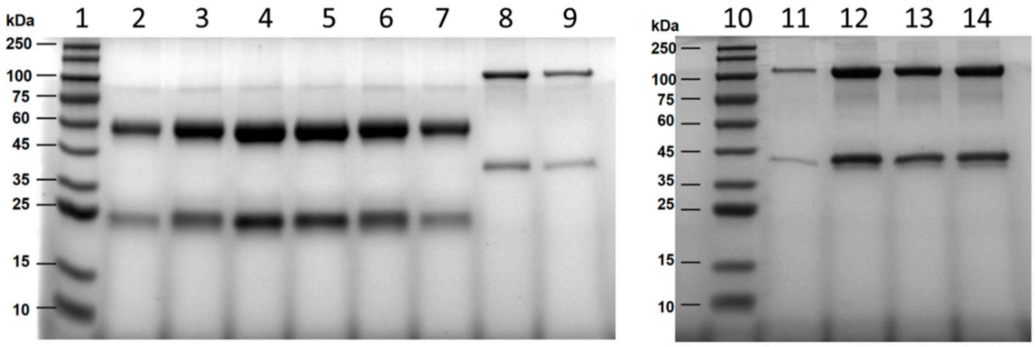


**Figure S1. SDS-PAGE analysis of monoclonal antibodies iMab, PRO140, 10E8, PGT121, PGDM1400, VRC01, trispecific antibodies iMab+PRO140+10E8, iMab+PRO140+PGMD1400, and iMab+PRO140+PGT121 expression and purification.** Lane 1: Marker; Lane 2. iMab (5 μg); Lane 3. PRO140 (5 μg); Lane 4.10E8 (5 μg); Lane 5. PGMD1400 (5 μg); Lane 6. PGT121 (5 μg); Lane 7. VRC01 (5 μg); Lane 8. Trispecific antibody iMab+PRO140+10E8 (1 μg); Lane 9. Trispecific antibody iMab+PRO140+PGMD1400 (1 μg); Lane 10. iMab+PRO140+PGT121 (1 μg); Lane 11. Trispecific antibody iMab+PRO140+10E8 (5 μg); Lane 12. Trispecific antibody iMab+PRO140+PGMD1400 (5 μg); Lane 13. iMab+PRO140+PGT121 (5 μg).

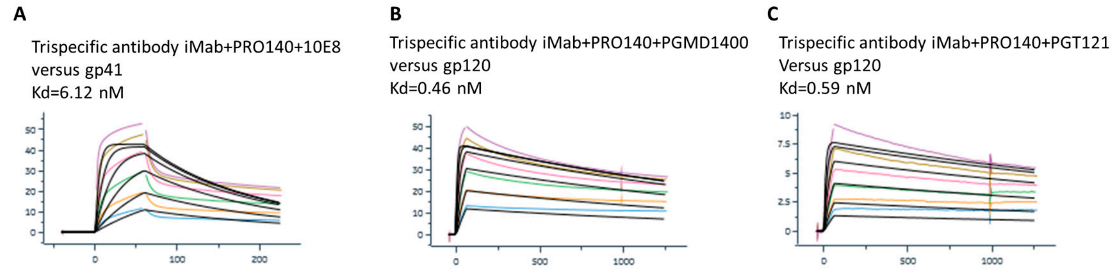


**Figure S2. Binding curves for reported Kd values for binding of tree trispecific antibodies to HIV-1 antigens.** The other curves are the experimental traces from testing various concentrations of antibodies in SPR experiments. (**A**) Trispecific antibody iMab+PRO140+10E8 versus gp41. (**B**) Trispecific antibody iMab+PRO140+PGMD1400 versus gp120. (**C**) Trispecific antibody iMab+PRO140+PGT121 versus gp120.

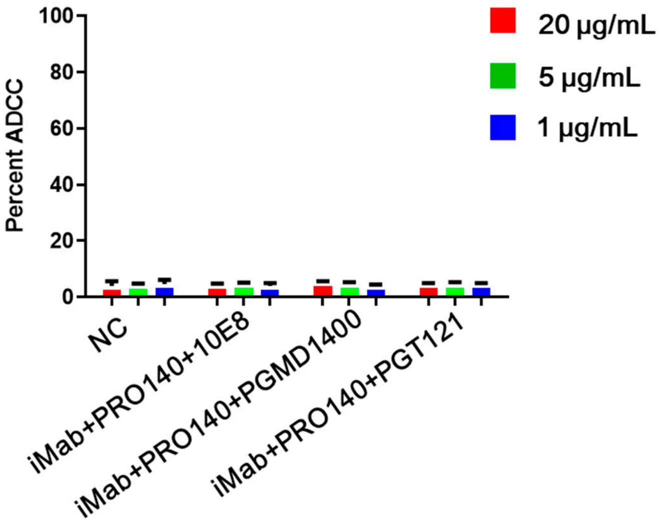


**Figure S3 Antibody-dependent cell-mediated cytotoxicity (ADCC) activity of three HIV-1 trispecific antibodies against CD4+ CCR5+ TZM-BL cells.** ADCC values for iMab+PRO140+10E8, iMab+PRO140+PGMD1400 and iMab+PRO140+PGMD1400 (1, 2, and 20 μg/ml) against TZM-BL cell, data represent the mean ± SEM.at 20 μg/mL, 2 μg/mL and 1 μg/mL.

**Table S1. Summary of monoclonal antibodies and their recognition targets used in this study.**

| Antibody | Target |
|---|---|
| iMab | CD4 receptor |
| PRO140 | CCRS co-receptor |
| 10E8 | Membrane-proximal external region of gp41 of HIV |
| PGMD1400 | V2 apex of HIV |
| PGT121 | N332 glycan supersite of HIV |
| VRC01 | CD4 binding site of HIV |

**Table S2. Statistical analysis (t-test) of IC50 values for neutralizing HIV-1 in each antibody.** *p < 0.05, **p < 0.05, ***p < 0.001, ****p < 0.0001.

| T-test | Significance | P value summary | P value |
|---|---|---|---|
| iMab vs. PRO140 | No | ns | 0.2246 |
| iMab vs. 10E8 | Yes | * | 0.0243 |
| iMab vs. PGDM1400 | No | ns | 0.127 |
| iMab vs. PGT121 | Yes | ** | 0.0047 |
| iMab vs. iMab+ PRO140+10E8-Cocktail | Yes | **** | <0.0001 |
| iMab vs. iMab+ PRO140+PGDM1400-Cocktail | Yes | **** | <0.0001 |
| iMab vs. iMab+ PRO140+ PGT121-Cocktail | Yes | **** | <0.0001 |
| iMab vs. iMab+ PRO140+10E8 | Yes | **** | <0.0001 |
| iMab vs. iMab+ PRO140+PGDM1400 | Yes | **** | <0.0001 |
| iMab vs. iMab+ PRO140+ PGT121 | Yes | **** | <0.0001 |
| iMab vs. VRC01 | Yes | ** | 0.0063 |
| PRO140 vs. 10E8 | No | ns | 0.2842 |
| PRO140 vs. PGDM1400 | No | ns | 0.998 |
| PRO140 vs. PGT121 | No | ns | 0.0937 |
| PRO140 vs. iMab+ PRO140+10E8-Cocktail | Yes | **** | <0.0001 |
| PRO140 vs. iMab+ PRO140+PGDM1400-Cocktail | Yes | **** | <0.0001 |
| PRO140 vs. iMab+ PRO140+ PGT121-Cocktail | Yes | **** | <0.0001 |
| PRO140 vs. iMab+ PRO140+10E8 | Yes | **** | <0.0001 |
| PRO140 vs. iMab+ PRO140+PGDM1400 | Yes | **** | <0.0001 |
| PRO140 vs. iMab+ PRO140+ PGT121 | Yes | **** | <0.0001 |
| PRO140 vs. VRC01 | No | ns | 0.1154 |
| 10E8 vs. PGDM1400 | No | ns | 0.4499 |
| 10E8 vs. PGT121 | No | ns | 0.537 |
| 10E8 vs. iMab+ PRO140+10E8-Cocktail | Yes | **** | <0.0001 |
| 10E8 vs. iMab+ PRO140+PGDM1400-Cocktail | Yes | **** | <0.0001 |

| | | | |
|---|---|---|---|
| 10E8 vs. iMab+ PRO140+ PGT121-Cocktail | Yes | **** | <0.0001 |
| 10E8 vs. iMab+ PRO140+10E8 | Yes | **** | <0.0001 |
| 10E8 vs. iMab+ PRO140+PGDM1400 | Yes | **** | <0.0001 |
| 10E8 vs. iMab+ PRO140+ PGT121 | Yes | **** | <0.0001 |
| 10E8 vs. VRC01 | No | ns | 0.6079 |
| PGDM1400 vs. PGT121 | No | ns | 0.172 |
| PGDM1400 vs. iMab+ PRO140+10E8-Cocktail | Yes | **** | <0.0001 |
| PGDM1400 vs. iMab+ PRO140+PGDM1400-Cocktail | Yes | **** | <0.0001 |
| PGDM1400 vs. iMab+ PRO140+ PGT121-Cocktail | Yes | **** | <0.0001 |
| PGDM1400 vs. iMab+ PRO140+10E8 | Yes | **** | <0.0001 |
| PGDM1400 vs. iMab+ PRO140+PGDM1400 | Yes | **** | <0.0001 |
| PGDM1400 vs. iMab+ PRO140+ PGT121 | Yes | **** | <0.0001 |
| PGDM1400 vs. VRC01 | No | ns | 0.8776 |
| PGT121 vs. iMab+ PRO140+10E8-Cocktail | No | ns | 0.227 |
| PGT121 vs. iMab+ PRO140+PGDM1400-Cocktail | No | ns | 0.1935 |
| PGT121 vs. iMab+ PRO140+ PGT121-Cocktail | No | ns | 0.2048 |
| PGT121 vs. iMab+ PRO140+10E8 | Yes | **** | <0.0001 |
| PGT121 vs. iMab+ PRO140+PGDM1400 | Yes | **** | <0.0001 |
| PGT121 vs. iMab+ PRO140+ PGT121 | Yes | **** | <0.0001 |
| PGT121 vs. VRC01 | No | ns | 0.2066 |
| iMab+ PRO140+10E8-Cocktail vs. iMab+ PRO140+PGDM1400-Cocktail | Yes | ** | 0.0018 |
| iMab+ PRO140+10E8-Cocktail vs. iMab+ PRO140+ PGT121-Cocktail | Yes | ** | 0.0088 |
| iMab+ PRO140+10E8-Cocktail vs. iMab+ PRO140+10E8 | No | ns | 0.167 |
| iMab+ PRO140+10E8-Cocktail vs. iMab+ PRO140+PGDM1400 | Yes | **** | <0.0001 |
| iMab+ PRO140+10E8-Cocktail vs. iMab+ PRO140+ PGT121 | Yes | **** | <0.0001 |
| iMab+ PRO140+10E8-Cocktail vs. VRC01 | Yes | **** | <0.0001 |

| | | | |
|---|---|---|---|
| iMab+ PRO140+PGDM1400-Cocktail vs. iMab+ PRO140+ PGT121-Cocktail | No | ns | 0.5834 |
| iMab+ PRO140+PGDM1400-Cocktail vs. iMab+ PRO140+10E8 | No | ns | 0.0683 |
| iMab+ PRO140+PGDM1400-Cocktail vs. iMab+ PRO140+PGDM1400 | Yes | *** | 0.0002 |
| iMab+ PRO140+PGDM1400-Cocktail vs. iMab+ PRO140+ PGT121 | Yes | * | 0.0295 |
| iMab+ PRO140+PGDM1400-Cocktail vs. VRC01 | Yes | **** | <0.0001 |
| iMab+ PRO140+ PGT121-Cocktail vs. iMab+ PRO140+10E8 | No | ns | 0.1973 |
| iMab+ PRO140+ PGT121-Cocktail vs. iMab+ PRO140+PGDM1400 | Yes | **** | <0.0001 |
| iMab+ PRO140+ PGT121-Cocktail vs. iMab+ PRO140+ PGT121 | Yes | ** | 0.0071 |
| iMab+ PRO140+ PGT121-Cocktail vs. VRC01 | Yes | **** | <0.0001 |
| iMab+ PRO140+10E8 vs. iMab+ PRO140+PGDM1400 | Yes | **** | <0.0001 |
| iMab+ PRO140+10E8 vs. iMab+ PRO140+ PGT121 | Yes | **** | <0.0001 |
| iMab+ PRO140+10E8 vs. VRC01 | Yes | **** | <0.0001 |
| iMab+ PRO140+PGDM1400 vs. iMab+ PRO140+ PGT121 | No | ns | 0.0818 |
| iMab+ PRO140+PGDM1400 vs. VRC01 | Yes | **** | <0.0001 |
| iMab+ PRO140+ PGT121 vs. VRC01 | Yes | **** | <0.0001 |
